# The influence of stress and social defeat on neurobiological reinforcement mechanisms across reward to withdrawal in nicotine addiction

**DOI:** 10.1007/s00213-025-06852-5

**Published:** 2025-07-26

**Authors:** Kokila Shankar, Sélène Zahedi, Olivier George

**Affiliations:** 1 Department of Psychiatry, University of California San Diego School of Medicine, 3272 Skaggs Pharmaceutical Sciences Building, 9500 Gilman Dr, La Jolla, CA 92093 USA; 2 Sanford Burnham Prebys Medical Discovery Institute, La Jolla, CA 92037 USA; 3 Institut de Neurosciences de la Timone, Aix-Marseille Université, 13005 Marseille, France

**Keywords:** Withdrawal, CRF, VTA, Dopamine, NAChR, Stress

## Abstract

Nicotine and cigarette/tobacco use continue to be a prevalent public health issue worldwide. The transition to nicotine addiction occurs through an allostatic cycle involving the stages of binging/intoxication, withdrawal/negative affective states, and preoccupation/anticipation. This review focuses on the psychological, neurobiological, and molecular mechanisms contributing to the negative affective state during withdrawal from nicotine with an emphasis on stress and how social defeat stress can affect these mechanisms. Psychologically, negative affect during withdrawal is thought to contribute to the transition from positive reinforcement of drug-taking to negative reinforcement of nicotine use. Nicotine binding to nicotinic acetylcholine receptors elicits a variety of neuronal signaling throughout the brain, over time producing within- and between-systems neuroadaptations across brain regions that govern reward, anxiety, pain, and stress responses. Continued nicotine use additionally dysregulates myriad molecular signaling pathways that directly affect nicotine intake/aversion and withdrawal-like symptoms. Throughout all of these mechanisms, non-pharmacological stress also plays an important role in mediating much of the negative affect associated with addiction. Social defeat stress increases a variety of neuropeptide signaling that consequently exacerbates drug taking and negative affective states. Understanding the mechanisms through which these stages manifest can better our understanding of addiction disease biology and provide novel avenues for therapeutic targets.

## Introduction

Despite increased policy and advocacy efforts over the decades, tobacco use remains the leading cause of preventable death and disease in the United States. Not only do almost half a million people die annually from tobacco use disorder (CDCTobaccoFree 2021), but cigarette smoking is also a significant factor in deaths caused by other respiratory diseases like lung cancer and chronic obstructive pulmonary disease (Current Cigarette Smoking Among Adults — United States 2005). Furthermore, cigarette smoking and tobacco use disorder cost the country $289 billion per year in associated healthcare costs and productivity loss (Key Substance Use and Mental Health Indicators in the United States: Results from the 2022 National Survey on Drug Use and Health 2022). In an effort to curb many of the negative health outcomes associated with tobacco use, electronic cigarette use has grown rapidly in recent years. While adolescents and young adults often view these as safer alternatives due to the lack of carcinogens like tar that are produced by traditional cigarettes, electronic cigarettes still pose significant risks to lung and cardiovascular health (Farsalinos and Polosa 2014; Hua and Talbot 2016) in the non-smoking population. E-cigarettes are also more popular among adolescents compared to traditional cigarettes, and both human and rodent studies have shown that initiation of e-cigarette use in adolescence can potentiate cigarette/tobacco use in adulthood (Barrington-Trimis et al. 2016; Kallupi et al. 2019).

However, the largest outcome of using these products is nicotine addiction. Nicotine is the primary psychoactive component of tobacco and is the motivating factor for people to use both traditional tobacco products and e-cigarettes (Foll et al. 2022; Markou 2008). Nicotine addiction can be defined as “a chronic, relapsing disorder that has been characterized by a compulsion to seek and take nicotine, loss of control over nicotine intake, and emergence of a negative emotional state (*e.g.*, dysphoria, anxiety, and/or irritability) that defines a motivational withdrawal syndrome when access to nicotine is prevented” (Koob and Moal 2008). This is summarized as the now established three-stage cycle, comprising phases of binge/intoxication, withdrawal/negative affect, and preoccupation/anticipation (Koob 2011). However, this cycle is allostatic – rather than maintaining homeostasis in brain circuitry, over time this cycle worsens; the brain must constantly work to operate at lower hedonic affective set points (Koob and Moal 2001; Koob and Schulkin 2019). This allostasis also represents the transition from drug taking as positive reinforcement to drug taking as negative reinforcement to avoid or mitigate the negative affective state. Factors outside of nicotine intake can also serve as positive and negative reinforcers that contribute to the emergence of the negative affective state. The social aspects of smoking like social bonding or imitating loved ones, for example, can serve as a positive reinforcer of nicotine use (Urbán 2010; Vink et al. 2003; NIDA Research Monograph 1976). Non-pharmacological stressors, including social defeat, stressful situations, or environmental stimuli, can serve as powerful negative reinforcement of smoking behavior (Ansell et al. 2012; Sinha 2008).

A critical goal in studying nicotine addiction is to identify new avenues to aid nicotine cessation in patients. Less than 30% of smokers who quit use FDA-approved medication, highlighting the need for increased awareness and access to these medications. Current therapeutics for nicotine cessation are effective, with varenicline being the most effective FDA-approved cessation therapy, doubling or tripling the odds of quitting after a year (Shang et al. 2023; Ebbert et al. 2015). However, long-term abstinence remains a challenge, and only 25% of smokers remain abstinent after 1 year, highlighting the need to improve current treatment strategies. Understanding the factors that drive nicotine withdrawal and negative affect can allow for more efficacious therapeutic development. The primary goal of this review is to examine psychological, neurobiological, and molecular contributions to the negative affective state during nicotine addiction. This review will also touch upon the role of non-pharmacological stress in initiating and potentiating the affective states of nicotine addiction.

## Transition from positive to negative reinforcement

Reinforcement plays a key role in nicotine addiction as many behaviors are due to responses to specific stimuli, or reinforcers, that increase the probability of these behaviors – in this case, drug taking. Positive reinforcement is defined as the probability of a response or behavior increasing following presentation of a stimulus; conversely, negative reinforcement occurs when the probability of a response is increased through removal of an aversive stimulus (Koob et al. 2014). Nicotine serves as the positive reinforcing stimulus, and the negative affective state during nicotine withdrawal serves as the negative reinforcing stimulus (Watkins et al. 2000; George and Koob 2017).

Nicotine, compared to other drugs of abuse, has a much narrower range of doses that are considered appetitive. Even moderate doses can be aversive or punishing, especially in nondependent subjects (Fowler and Kenny 2014; Koffarnus and Winger 2015). Acute nicotine intake produces increased anxiogenic-like behaviors, increases in the circulating stress biomarkers adrenocorticotropin-releasing hormone and cortisol, and unpleasant physiological responses like cough or nausea (Casarrubea et al. 2015; Newhouse et al. 1990). The severity of these aversive reactions has been shown to affect the development of chronic nicotine use, with those experiencing more aversive effects after first use less rapidly transitioning to regular use (Sartor et al. 2010). In preclinical models of self-administration, nicotine doses are carefully titrated to prevent nicotine from serving as an aversive stimulus (Donny et al. 1998). Through developing tolerance to the noxious effects of nicotine, habitual use can be established (Fowler and Kenny 2014; Russell 1979).

### Nicotine taking as positive reinforcement

While acute nicotine initially produces aversive responses, it also produces acutely pleasurable effects on mood and cognition in humans, including mild euphoria, increased energy and focus, and increased arousal (Pomerleau and Pomerleau 1992a; Stolerman and Jarvis 1995; Benowitz 1996). These positive effects can increase the likelihood of users repeatedly consuming nicotine. In fact, it was shown in a trial of smokers that subjects displayed greater positive reinforcement learning following cigarette consumption (Baker et al. 2020). The acute positive reinforcing effects of nicotine are important in establishing self-administration behavior. Smoking is also used for relaxation in response to stress (Benowitz 1988). Evidence from human and non-human animal studies points to various stressors throughout early life, adolescence, and adulthood as contributors to increased drug abuse either through earlier initiation of use, faster transition to increased use and/or binge patterns, and increased positive reinforcement of substance use (Torres and O’Dell 2016; Kirsch and Lippard 2022; Ahmed et al. 2020).

Rodent models of intravenous nicotine self-administration have been reliably used for decades as they elicit positive reinforcement that consequently produces increased intake over time (Corrigall 1992; Donny et al. 1995). This robust increase in intake is seen especially in models that use intermittent extended access to nicotine self-administration, leading to increased motivation for drug-taking (Cohen et al. 2012). It has been shown in both human and rodent tests that increased nicotine reinforcement is strongly positively correlated with cue-induced craving (humans) and cue-induced reinstatement (rodents) (Butler et al. 2021).

The social defeat stress model pioneered by Dr. Klaus Miczek has become another valuable tool to study the contribution of stress on drug-taking and positive reinforcement, serving as a method of “behavioral sensitization” to stimulants or other drugs of abuse (Covington and Miczek 2001). Miczek and colleagues’ seminal studies in this field have shown that intermittent and chronic stress exposure through this model has persistent effects on binge intake of the psychostimulant cocaine, shortens the latency to acquire drug, and increases motivation for drug (Covington and Miczek 2001; Covington et al. 2005; Miczek et al. 2004). This model of social defeat stress has been adapted to investigate nicotine use, and interestingly, studies using this model have found that nicotine administered after exposure to social defeat stress may actually confer some resilience to stress in rodents (Parise et al. 2020; Zou et al. 2014). Throughout this review, we will incorporate the role of social defeat stress and external stressors in perpetuating the negative affective state and related behaviors.

### Nicotine taking as negative reinforcement

**Fig. 1:**
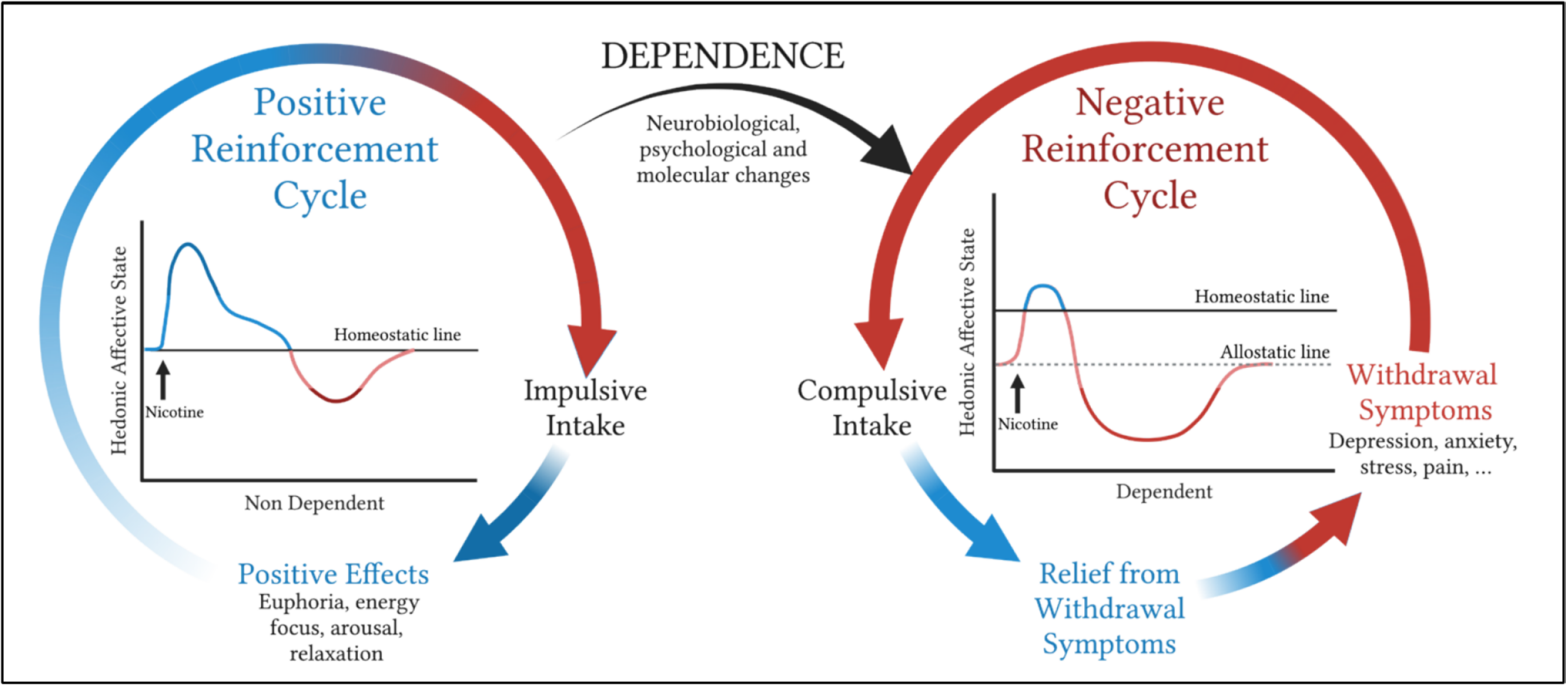
Transition from positive to negative reinforcement of nicotine. Initial nicotine use produces a cycle of positive reinforcement (left) characterized by positive behavioral effects (blue) that consequently leads to impulsive nicotine intake. Abstinence from nicotine elicits a transient negative affective state (red). Repeated nicotine use prompts a downward shift of the hedonic set point, resulting in allostasis where the negative affective state is consistent. Thus, drug use shifts to compulsive intake to avoid negative states

Nicotine may be exerting its addictive effects through the transition from positive to negative reinforcement, as well as the shift from impulsive to compulsive behavior (Fig. 1). Impulsivity is defined as “a predisposition toward rapid, unplanned reactions to internal and external stimuli without regard for the negative consequences of these reactions to themselves or others” (Moeller et al. 2001). Impulsivity is present at the early stages of addiction, and it is thought to be produced by positive reinforcement mechanisms. Feelings of intense arousal and excitement prior to the stimulus, as well as feelings of euphoria, reward, and pleasure after the behavior is performed, are the primary drivers of positive reinforcement (Perkins et al. 2003; Pomerleau and Pomerleau 1992b). Smokers have been shown to exhibit increased impulsive intake of nicotine compared to nonsmokers (Mitchell 2004; Field et al. 2006). However, there are often feelings of guilt or regret following the behavior, as drugs are normally taken for their initial pleasurable effects but potential negative consequences are not taken into account (Yang et al. 2019). Over time, behavioral and neural adaptations shift drug taking to a negative reinforcement mechanism. Anxiety, stress, and pain prior to the behavior promote negative reinforcement, and once the behavior is performed, individuals experience relief from the negative emotional state (Pang et al. 2014). Compulsivity, defined as “perseverative, repetitive actions that are excessive and inappropriate,” takes over from impulsivity in the later stages of addiction (Berlin and Hollander 2014). This compulsivity has been historically thought to drive escalation of drug intake despite adverse consequences, although compulsivity as a measure is far more complex and cannot provide the whole picture of negative reinforcement in addiction (George et al. 2022).

Chronic nicotine use produces robust withdrawal or negative affective behaviors in both humans and nonhuman animal models (Chellian et al. 2021; Hamilton et al. 2009; Hughes et al. 1994; Malin and Goyarzu 2009; Stoker et al. 2008; O’dell et al. 2004). Reported in the DSM-V, acute symptoms in humans include depression, anxiety, irritability, dysphoria, stress, as well as somatic symptoms like bradycardia and increased appetite; in protracted abstinence, users can experience longer-lasting symptoms of depressed mood, irritability, increased stress (McLaughlin et al. 2015; Diagnostic and Statistical Manual of Mental Disorders n.d.). In rodent models, physical symptoms of nicotine withdrawal are characterized by somatic signs like body shakes, ptosis, teeth chattering, etc (Ponzoni et al. 2015; Damaj et al. 2003; Grabus et al. 2005). Social defeat stress can facilitate these symptoms, including nicotine-induced locomotor suppression (Domingues et al. 2019). Withdrawal also induces changes in motivation as measured by behavioral tests like intracranial self-stimulation and conditioned place aversion, and importantly changes in affect as seen using open-field tests that measure anxiety-like behaviors, depression-like behaviors, and anhedonia-like behaviors (Epping-Jordan et al. 1998; Kim and Im 2024; Torres et al. 2013; Wang et al. 2018; Jackson et al. 2013; Bruijnzeel and Markou 2004). These behaviors are most often observed in models of extended access and/or chronic use of passive or self-administered nicotine *vs.* acute intake (Cohen and George 2013). We have observed that within a novel rodent model of nicotine vapor self-administration, 3 weeks of 1 h self-administration sessions was sufficient to produce increased somatic signs of withdrawal and hyperalgesia precipitated by mecamylamine-induced withdrawal, as well as anxiety-like behaviors following protracted abstinence, similar to effects seen in both human smokers and intravenously self-administering rodents (Smith et al. 2020). In these models, an abstinence period of just 2–3 days is sufficient to trigger withdrawal-like behaviors in rodents, and consequently increase self-administration of nicotine when access to drug is restored (Cohen et al. 2012; O’dell et al. 2007). This finding mimics human behavior, as after an abstinence period, people smoke more (Isaac and Rand 1972; Nil et al. 1987; Rusted et al. 1998).

It is believed that withdrawal symptoms drive the shift to negative reinforcement – the motivation for nicotine intake changes to avoid these negative affective behaviors, rather than to seek out pleasurable effects. In fact, it has been shown that negative affective symptoms of anxiety-like behavior and hyperalgesia induced by nicotine abstinence predicted subsequent nicotine intake when access was restored (Cohen et al. 2015). In human studies, there is a similar link between negative affect and smoking, as assessed by a meta-analysis of clinical data (Akbari et al. 2020). This data shows that severity of these behaviors is a key factor in dependent animals’ excessive nicotine intake. While we have described the transition from positive to negative reinforcement from a psychological lens, negative affect can additionally be explained by neurobiological and molecular changes in the mechanisms of motivation.

## Neurobiological mechanisms of negative affect

Nicotine’s mechanism of action comes from binding to neuronal nicotinic acetylcholine receptors (nAChRs), which are present ubiquitously in various conformations throughout the brain. Specifically, neuronal nAChRs are heteropentameric proteins consisting of various conformations of alpha- and beta-subunits (α2-α10 and β2-β4) (Changeux et al. 1998; Vidal and Changeux 1996), all of which produce different pharmacological properties in the brain. These receptors act as ligand-gated ion channels which primarily regulate endogenous acetylcholine signaling but also have affinity for exogenous nicotine (Changeux 2010). When nicotine binds to these receptors, there is increased permeability of ions which serve to depolarize the cell and lead to neuronal firing.. nAChRs, and specific subunits, play critical roles in all components of addiction, from positive reinforcement to negative affect, and nAChR subunits have even been shown to mediate both positive and negative effects of nicotine. The α4, α5, α6, and β2 subunit-containing receptors are critical for mediating the positive reinforcing effects of nicotine (Changeux 2010; Jackson et al. 2008; Picciotto et al. 1998; Wigestrand et al. 2011; Brunzell et al. 2015). Continued nicotine use also leads to upregulation of nAChRs due to receptor desensitization by nicotine (Henderson and Lester 2015).

### The mesocorticolimbic system as a positive reinforcer of nicotine intake

The reinforcing effects of nicotine are primarily driven by the mesocorticolimbic system, comprising the basal ganglia (including the nucleus accumbens (NAc) and striatum) and ventral tegmental area (VTA). Within the VTA, nAChRs are found on dopamine-producing neurons; when stimulated by nicotine, these cells release dopamine to downstream sites including the NAc, striatum, and prefrontal cortex (Xiao et al. 2020; Faure et al. 2014). Dopamine release is an important mechanism that enhances and sustains the motivation for and reinforcement of drug-taking behavior (Volkow et al. 2017). With continued nicotine use, dopaminergic neurons become desensitized and have reduced firing during withdrawal, which contributes to the negative affective state (Wills et al. 2022). Stress has been shown to activate dopaminergic signaling in the VTA and lead to increased drug-taking in rodent models of multiple drugs of abuse, including nicotine (Holly and Miczek 2016; Morel et al. 2018; Ortiz et al. 2022; Adams et al. 2021). Furthermore, they showed that social defeat stress alters mesocorticolimbic dopamine release that activates a VTA-accumbens-prefrontal cortex-amygdala neural circuit (Miczek et al. 2008; Yap and Miczek 2008). Both acute stressors and repeated stress exposure can induce persistent neuroadaptations in VTA dopaminergic signaling that can consequently alter addiction-like behaviors (Holly and Miczek 2016; Tidey and Miczek 1996).

### nAChR signaling within withdrawal and negative affect

nAChRs mediate the physical and affective components of nicotine withdrawal differently. The β2 subunit is necessary for the anxiogenic and aversive effects of nicotine withdrawal, as demonstrated by β2 knockout mice exhibiting losses of anxiety-like behaviors, conditioned place aversion, and withdrawal-induced deficits in contextual fear conditioning (Jackson et al. 2008; Jackson et al. 2009a; Portugal et al. 2008). The α6 subunit also plays a role in the anxiety-like symptoms of nicotine withdrawal (Jackson et al. 2009b). The α3β4 receptor and α5 subunit independently mediate physical signs of withdrawal, as shown by a decrease in somatic signs of withdrawal and hyperalgesia following selective blockade of these receptors (Jackson et al. 2013). The α7 receptor, which exhibits more rapid desensitization upon nicotine binding, is involved in producing somatic signs of withdrawal. Knockout of α7 subunits has been shown to delay self-stimulation (Stoker et al. 2008; Salas et al. 2007), potentially also giving it a role in the anhedonic behaviors associated with negative affect during withdrawal. α7 increases sensitivity to social defeat stress in female rats, which may increase vulnerability to addiction (Ortiz et al. 2022). Additional information about the role of nAChR subtypes in mediating different aspects of nicotine addiction can be found in a comprehensive review by Picciotto and Kenny (2021).

Given the wide prevalence of nAChRs across most brain regions, other neurotransmitter systems are also involved in contributing to positive reinforcement and/or negative affective behaviors. Acetylcholine is the primary endogenous ligand for neuronal nAChRs. Acetylcholine as a neuromodulator is known to increase release of additional neurotransmitters, promote or suppress neuronal firing, and contribute to synaptic plasticity (Picciotto et al. 2012). Endogenous acetylcholine signaling has been shown to activate the dopaminergic signaling system, similar to exogenous nicotine (Maskos 2010; Clarke et al. 1987; Zhou et al. 2001). It was shown that upon administration of a positive allosteric modulator for the α7 nAChR subunit, endogenous acetylcholine potentiation was sufficient to attenuate precipitated nicotine withdrawal as measured by somatic signs of withdrawal in mice (Jackson et al. 2018).

### The extended amygdala

**Fig. 2:**
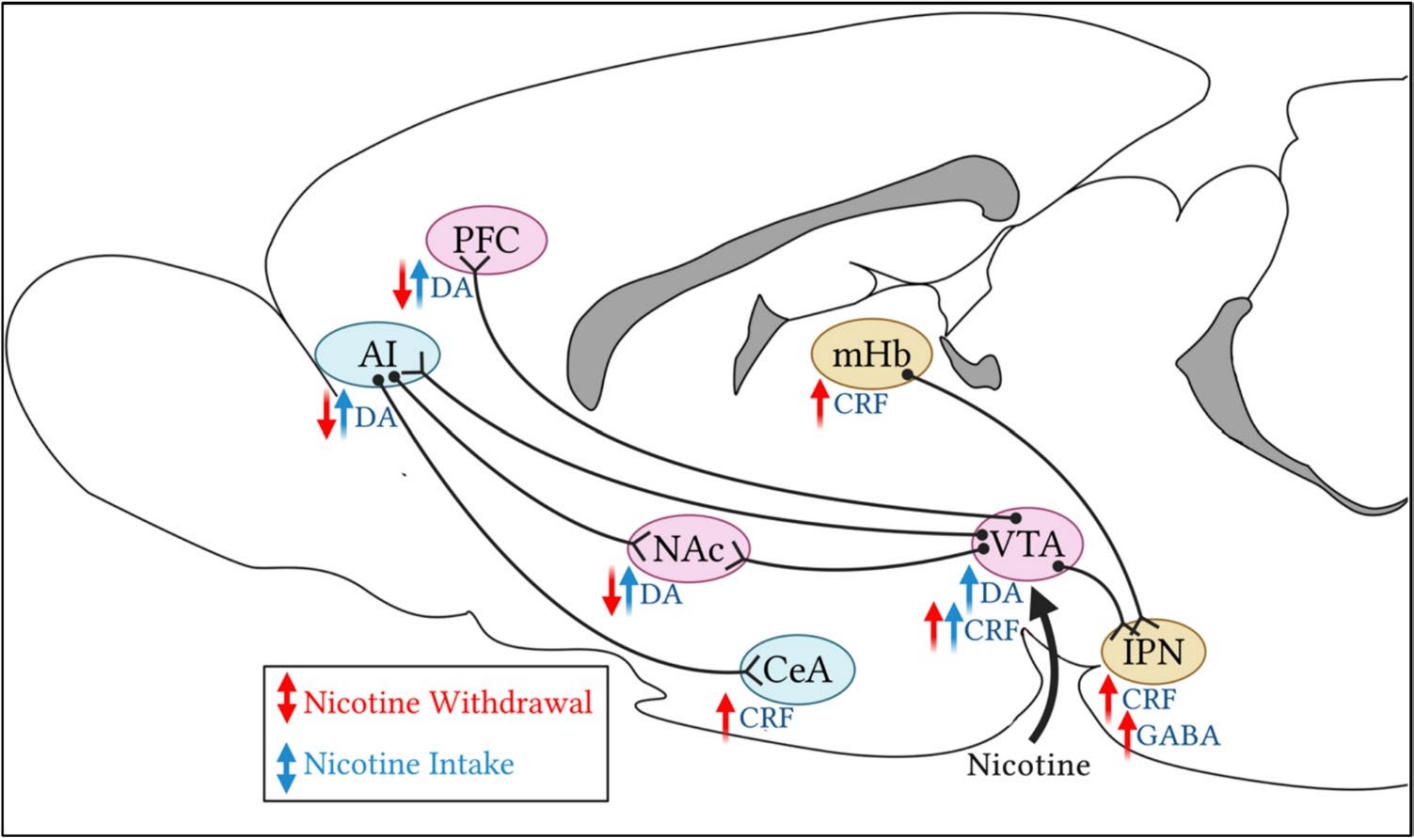
Neurobiological mechanisms involved in negative affect during nicotine withdrawal. Sagittal rat brain outline showing neurocircuitry and transmitter signalling. Nicotine intake increases dopamine (DA) in the mesocorticolimbic dopamine system (ventral tegmental area (VTA), nucleus accumbens (NAc), and prefrontal cortex (PFC); pink) and the anterior insula (AI); however, prolonged use decreases DA, impacting reward function and exacerbating withdrawal symptoms and craving. Nicotine also triggers corticotropin-releasing factor (CRF) signaling in the VTA to enhance reward sensitization, and in the central nucleus of the amygdala (CeA), medial habenula (mHb), and interpeduncular nucleus (IPN), influencing withdrawal circuits and contributing to anxiety-like behaviors and negative emotional states. Additionally, VTA neurons activate IPN GABA (γ-aminobutyric acid) neurons, heightening anxiety-like behaviors

With continued nicotine use, within-system neuroadaptations occur which produce a decrease in dopamine signaling within the mesocorticolimbic pathway, leading to the overall reduction in brain reward function that may contribute to the negative affect stage of the addiction cycle. Along with within-system neuroadaptations, between-system neuroadaptations play an important role in the development of the negative affective state. The primary circuit involved in withdrawal and negative affect is the extended amygdala, consisting of the central nucleus of the amygdala (CeA), bed nucleus of the stria terminalis (BNST), and a portion of the NAc shell (Koob and Volkow 2010) (Fig. 2). These brain regions are known to play a key role in emotional processing and stress-related behaviors. Mineur et al*.* showed that within the amygdala, both α7 and β2 subunits were important for different stress-mediated behaviors, with α7 knockout mice displaying reduced anxiety-like and depressive-like behaviors, and β2 knockout mice exhibiting decreased resistance to social defeat stress (Mineur et al. 2016). Chronic nicotine exposure has been shown to facilitate long-term potentiation within the amygdala, and this requires α7 and β2 subunits (Huang et al. 2008).

### The anterior insular cortex

The anterior insular cortex, with afferents to the NAc and extended amygdala and dopaminergic terminals from the VTA, is another prime candidate influenced by nicotine addiction (Ibrahim 2019). Nicotine has been shown to activate the insular cortex, and damage to this region has been shown to lead to reduced withdrawal symptoms, craving for nicotine, and even relapse (Naqvi et al. 2007). The insula is believed to integrate sensory and cognitive signals as well as modulate the salience network. During acute abstinence from nicotine, studies have shown that the insula becomes flooded with craving signals, and signaling to downstream networks is enhanced, suggesting the insula’s role in producing the motivation to obtain the drug within the context of negative reinforcement (Naqvi and Bechara 2009; Lerman et al. 2014; Regner et al. 2019). Imaging studies looking at the function of the insula during craving have reported increased activation in chronic nicotine users when presented with cues, and smoking consequently decreased connectivity of the insula to the anterior cingulate cortex (Goudriaan et al. 2010; Faulkner et al. 2019). While studies have not explored the direct role of CRF signaling within the insula in mediating negative affect in nicotine addiction, CRF signaling within the insula is known to mediate other types of addiction (Goudriaan et al. 2010; Rotge et al. 2017; Sánchez et al. 1999; Martin-Fardon et al. 2010; Cottone et al. 2009).

### The habenula-interpeduncular nucleus circuit

Another circuit highly susceptible to nAChR signaling is the habenular-interpeduncular nucleus axis. The habenula is divided into the lateral habenula and medial habenula, which exhibit different actions within nicotine addiction. The lateral habenula contains primarily α6 nAChR subunits, and it receives inputs from the VTA and sends outputs to dopaminergic neurons; activation of the lateral habenula decreases downstream dopaminergic signaling, and following chronic nicotine, this activation is no longer present, suggesting a role for the lateral habenula in regulating reward-related activity (Pierucci et al. 2022; Matsumoto and Hikosaka 2007; Paolini and Biasi 2011; Zuo et al. 2016). The medial habenula projects primarily to the interpeduncular nucleus (IPN). This circuit is densely populated with α3, α5, and β4 nAChR subunits that were shown above to be necessary for the physical symptoms of nicotine withdrawal, suggesting that this pathway may be mediating withdrawal-like behaviors (Salas et al. 2009; Fowler et al. 2011). The medial habenula has been shown to regulate aversion to nicotine, including through expression of the β4 subunit (Frahm et al. 2011; Elayouby et al. 2021; Ślimak 2014). Medial habenular α5 nAChR subunits also regulate nicotine intake, as Fowler *et. al* found that α5 knockdown in mice in this region dose-dependently inhibited brain reward thresholds, and subsequent disruption of the IPN in these models led to increased nicotine intake (Fowler et al. 2011). In the IPN, expression of the α2 subunit was shown to be important for fear conditioning, another affective symptom of withdrawal (Ishii et al. 2005; Lotfipour et al. 2013). Within the IPN, these withdrawal-like behaviors are mediated by GABAergic neuron activation specifically induced by nicotine withdrawal in dependent animals (Avelar and Gearge 2022; Zhao-Shea et al. 2013).

## Between-systems neuroadaptations within negative affect

### Corticotropin*-*releasing factor and stress signaling

Along with within-system neuroadaptations, between-system neuroadaptations play a large role in the shift to negative reinforcement. One of these between-system neuroadaptations is the extrahypothalamic stress system. Nicotine activates the hypothalamic–pituitary–adrenal (HPA) axis – the major stress signaling pathway in the brain and body – and causes the subsequent release of corticotropin-releasing factor (CRF) from the hypothalamus (Matta et al. 1998; Okada et al. 2003). Acute nicotine has been shown to be a potent activator of the HPA axis, causing the elevation of brain CRF and circulating adrenocorticotropic hormone and cortisol/corticosterone (Matta et al. 1998; Steptoe and Ussher 2006). Reciprocally, acute stress has been widely anecdotally cited and shown in laboratory settings to decrease resistance to smoking and cause more intense nicotine use in regular smokers (McKee et al. 2011; Al’Absi 2006). Like other drugs of abuse, nicotine activates CRF signaling in many regions of the brain, and this overactivation of CRF signaling may be responsible for many of the stress-related behaviors seen in withdrawal. Nicotine withdrawal produces increases in extracellular CRF levels in the CeA, and antagonism of the CRF receptors has been shown to block anxiety-like behavior and deficit in brain reward function seen with precipitated nicotine withdrawal in rodents (George et al. 2007; Bruijnzeel et al. 2007; Heinrichs 2003; Heinrichs and Koob 2004). Since then, many studies have confirmed the role of CRF in withdrawal-like behaviors through various manipulation studies within the extended amygdala (Cohen et al. 2015; Bruijnzeel et al. 2007; Bruijnzeel et al. 2009; Qi et al. 2014; Qi et al. 2016).

The habenula-IPN pathway is also influenced by the CRF system (Grieder et al. 2014; Zhao-Shea et al. 2015). The IPN receives inputs from the VTA, and activation of this VTA-IPN circuit has been implicated in promoting anxiety-like behaviors through increased CRF signaling from the VTA (Zhao-Shea et al. 2015). Grieder et al*.* found that dopaminergic neurons in the VTA that also express CRF have been shown to upregulate expression of CRF encoding-mRNA following chronic nicotine exposure. Virus-mediated downregulation of CRF-encoding mRNA in the VTA, as well as pharmacological antagonism of CRF_1_ receptors within the IPN, not only prevented withdrawal-induced negative affective state, but also prevented increased nicotine intake following an abstinence period. Building upon this work, Zhao-Shea et al*.* identified a meso-interpeduncular circuit in which VTA CRF neurons innervate both medial habenula glutamatergic cells and CRF1-expressing IPN neurons, leading to increased anxiety during nicotine withdrawal in rodents (Zhao-Shea et al. 2015). We have further characterized the role of CRF in nicotine addiction in a previous review (Simpson et al. 2020).

External stress can also influence CRF signaling in the VTA. Miczek and colleagues have shown that social defeat stress elevates CRF levels in the VTA, increasing activation of dopaminergic neurons in the VTA and leading to heightened dopamine signaling in the NAc (Holly et al. 2015). This signaling cascade sensitizes the reward pathway, making rodents more prone to substance-seeking behavior (Holly et al. 2016; Leonard et al. 2017; Hwa et al. 2016; Albrechet-Souza et al. 2015). Antagonism of the CRF1 receptor selectively in the VTA blocked these CRF-induced effects and prevents the escalation of drug-seeking behavior seen after social stress (Han et al. 2017; Burke et al. 2016). CRF signaling in the VTA is a crucial mediator in the link between stress and vulnerability to substance use disorder, opening the door to target CRF signaling in the VTA as a potential therapeutic strategy.

### Opioid receptor signaling

Another critical between-system neuroadaptation altered by nicotine use is endogenous opioid signaling. The *mu* opioid receptor (MOR) is implicated in both positive reinforcement and negative affective behaviors through its regulation of behavioral sensitization and analgesia. Repeated social defeat stress has been shown to increase MOR expression in the VTA, and these receptors are located on GABAergic neurons; the subsequent decreased opioid signaling reduces inhibition of VTA dopaminergic neurons (Nikulina et al. 2008; Johnson and North 1992). MOR agonism following social defeat stress produces increased locomotor activity, further implicating a disinhibition of dopaminergic cells (Nikulina et al. 2005). MOR antagonism has been shown to elicit nicotine withdrawal symptoms in rodents (Biała et al. 2005; Malin et al. 1993), suggesting that this signaling system is needed for the reinforcing and rewarding effects of nicotine (Norman and D’Souza 2017). Dynorphin, the primary endogenous activator for the *kappa* opioid receptor (KOR), has been shown to increase in the brain following acute nicotine administration (Hadjiconstantinou and Neff 2011). This activation persists with chronic nicotine use and leads to increased anxiety-like behavior, decreased dopaminergic signaling, and increased receptor desensitization (Tejeda et al. 2012; McCarthy et al. 2010). KOR activation has also been shown to produce a stress-like state that may contribute to aversive affective behaviors and increase the propensity for reinstatement of drug-taking following abstinence (Wee and Koob 2010).

### Whole brain analysis to integrate signaling changes within nicotine withdrawal

Finally, while many of these canonical regions have been studied discretely or within limited circuits, the use of whole-brain analysis techniques allows for a more complete understanding of the brain-wide neurobiological shifts occurring during withdrawal. We have shown using iDISCO whole-brain clearing and FOS immunolabeling in mice that neural networks of healthy brains exhibit a high degree of modularity, but during nicotine withdrawal, brain connectivity increases to reduce the number of network modules to five key modules connecting almost every brain region. The largest modules are driven by cortical and extended amygdalar regions, which confirm that these regions drive the negative reinforcement and negative affect associated with withdrawal. We have further shown that these modularity changes correlate with increased expression of nAChR subunit genes and alteration of long-range cholinergic networks (Carrette et al. 2023). Understanding the changes to whole brain functional connectivity can guide more detailed analyses of brain regions, as well as identify previously unexplored regions that may be contributing to nicotine withdrawal.

## Molecular mechanisms of negative affect

Nicotine cessation strategies are variably effective, with approaches targeting the spectrum from nAChR desensitization to alleviating withdrawal symptoms. There are many behavioral treatments that try to target the rewarding effects of nicotine, either through decreasing the drug-related reward such as in cognitive behavioral therapy, or through increasing responses to natural reward such as in contingency management (Wardle et al. 2024). Additionally, now outdated strategies like rapid-smoking have tried to improve cessation through increasing aversion to nicotine intake (Hajek and Stead 2001; Tiffany et al. 1986). Current FDA-approved strategies target nicotine withdrawal or craving, focusing on alleviation of negative affective symptoms (Cohen et al. 2024). As there is a continued need to develop better therapeutics for nicotine cessation, many studies have shifted to obtaining a deeper understanding of molecular mechanisms governing the negative affective state within nicotine withdrawal. Identifying key contributors to the unpleasant behaviors experienced during withdrawal can provide insights into novel treatments for these symptoms and hopefully prevent or reduce relapse.

### Glucocorticoids

In mediating the stress response, the HPA axis relies on the production of CRF as well as circulating glucocorticoids (GCs), most notably cortisol in humans and corticosterone in rodents. As activation of the HPA axis is mediated by CRF, elevated CRF levels in the brain initiate a signaling cascade that ultimately leads to increased GC levels. Then, in a negative feedback loop, increased GC downregulates HPA axis signaling (Smith and Vale 2006; Gjerstad et al. 2018). In fact, GCs have been shown to regulate CRF expression (Tanimura and Watts 1998; Imaki et al. 1991). During chronic stress conditions, such as withdrawal following chronic nicotine use, circulating GC levels are decreased (Wong et al. 2014; Frederick et al. 1998). Conversely, increased GC administration can decrease some of the stress-related effects of sensitization to nicotine (Caggiula et al. 1998). These changes to GC signaling may be implicated in negative affect during nicotine withdrawal. It was shown that nicotine withdrawal in rats produced lower corticosterone levels during restraint stress, and the authors suggested that this may implicate the precipitation of depressive-like behaviors during nicotine abstinence (Semba 2004). GCs have also been shown to impact dopaminergic signaling: they can increase excitatory synaptic strength of dopaminergic synapses and promote desensitization of the reward system over time (De Jong 2004; Marinelli and Piazza 2002).

GCs are altered at both the extracellular and intracellular levels. Transcriptional profiling of the prefrontal cortex and NAc found that transcription factors associated with glucocorticoid receptors were significantly repressed in rats that underwent cocaine or oxycodone extended-access self-administration (Duttke et al. 2022). Interestingly, single nucleotide polymorphisms (SNPs) at the *FKBP5* gene have been shown to regulate severity of responses to nicotine withdrawal in humans (Jensen and Sofuoglu 2016). The *FKBP5* gene encodes a molecular co-chaperone of the GC receptor complex, so its activation is induced by GCs in order to terminate HPA axis signaling (Zannas et al. 2016). The SNP rs3800373*C, associated with a common *FKBP5* haplotype, yielded lower scores on the Minnesota Nicotine Withdrawal Scale in overnight abstinent smokers. Additional studies found similar results of this SNP in reducing alcohol withdrawal as well as a link between this SNP and heroin dependence (Huang et al. 2014; Levran et al. 2014). Altogether these studies show a link between the genetic regulation of glucocorticoids and the transition to substance abuse and negative affective behaviors. GC receptor antagonists are currently being investigated as treatments for alcohol use disorder, so there is potential for them to be investigated as treatments for nicotine use (McGinn et al. 2021; Vendruscolo et al. 2015).

### 5’ adenosine monophosphate kinase

Another molecular mechanism of interest has recently been shown to be the 5’ adenosine monophosphate kinase (AMPK) signaling pathway. AMPK is the key cellular regulator of energy metabolism; it assesses the ATP:AMP ratio and accordingly increases activity to generate ATP and ensure cells have the requisite amount of energy to function. AMPK is known to be necessary for synaptic activation, thus making it a crucial element of neuronal signaling (Marinangeli et al. 2018). Chronic nicotine use has been shown to increase energy metabolism through both increased locomotor activity and increased thermogenesis, thus producing a negative energy balance in the system that is due to hypothalamic AMPK becoming inactivated (Martínez de Morentin et al. 2012). Brynildsen et al*.* showed that activation of AMPK by AICAR (AMPK activator) and metformin (Type II diabetes therapeutic) abolished anxiety-like behaviors in mice during nicotine withdrawal. Furthermore, they saw that pretreating animals with systemic metformin prevented these anxiety-like behaviors while not impacting fed and fasted body weight, food intake, or glucose levels. Recently, similar results have been shown with overexpression of AMPK promoting antidepressant-like effects in mice undergoing withdrawal from methamphetamine, another psychostimulant (Hosseini et al. 2022). Impairment of AMPK generates cellular stress, which over time can lead to dysregulated signaling, oxidative stress, and even cell death (Liu and Chern 2015; Ronnett et al. 2009). Additional studies examining other metabolic disorder therapeutics such as glucagon-like peptide 1 (GLP-1) agonists have found these to similarly play a role in nicotine intake and aversion (Tuesta et al. 2017); we additionally have shown modulation of feeding-related hormone circulation following nicotine intake, suggesting a reciprocal relationship between nicotine and feeding regulation that could be explored for future therapeutic targets (Shankar et al. 2024). Energy metabolism clearly plays a role in modulating negative affective behaviors, but much work needs to still be done in this field to properly assess the mechanisms surrounding these effects.

### Brain-derived neurotrophic factor

Another protein of interest is brain-derived neurotrophic factor (BDNF), which is commonly known for its role in neuronal development and learning and memory. BDNF has been shown to play a role in nicotine dependence (Huang et al. 2021). Nicotine withdrawal following chronic treatment has been shown to increase BDNF levels in the NAc and VTA (Kivinummi et al. 2011). Increased BDNF expression has also been correlated with increased hyperalgesia during withdrawal (Shi et al. 2015). Recently, Grieder et al*.* showed that BDNF is necessary for the transition to nicotine dependence (Grieder et al. 2022). A single intra-VTA BDNF injection in drug-naïve mice produced a rewarding response to acute nicotine treatment and an aversive response to treatment 8 h after acute nicotine treatment similar to behavior seen in nicotine-dependent and nicotine-withdrawn mice, whereas control mice displayed opposite responses. The authors further showed that this behavior was due to a switch to dopamine receptor D2-mediated signaling, which they previously identified as occurring during nicotine withdrawal due to decreased tonic dopamine activity within the VTA. Rodent models of stress investigating the role of BDNF have similarly found that episodic stress increased BDNF levels in the VTA amplified dopaminergic signaling in the NAc, and these animals had increased psychostimulant intake (Miczek et al. 2011). However, continuous social defeat stress led to reduced VTA BDNF levels and increased anhedonia. Interestingly, in a mouse model of nicotine exposure through water pipe tobacco smoke or injection, chronic nicotine administration prior to stress promoted resilience to stress as seen by reduced social avoidance in the chronic social defeat stress paradigm (Khalifeh et al. 2020). BDNF signaling was found to confer this resilience as inhibition of the BDNF receptor tropomyosin receptor kinase B (TRKB) blocked the reduction in social avoidance after chronic stress. These studies suggest a critical role in BDNF signaling mediating the affective states of nicotine withdrawal.

### Cannabinoid signaling

Cannabinoid (CB) receptors, along with their endogenous cannabinoid ligands, are a broad signaling system with involvement in many physiological and psychological functions. One of these functions is a molecular mediator of addiction. Studies have examined the role of CBs in the rewarding properties of addictive substances, particularly due to the abundance of CBs in dopaminergic VTA neurons (Mátyás et al. 2008). In fact, CB receptor antagonism was shown to block the dopamine release within the NAc and the BNST (Cohen et al. 2002). The cannabinoid system has specifically been shown to regulate nicotine intake; studies have shown that CB1 receptor activation increases motivation to self-administer nicotine (Gamaleddin et al. 2015; Merritt et al. 2008). Conversely, blockade of CB1 prevents nicotine-induced conditioned place preference and increases in locomotor activity, and CB1 antagonism was shown to reduce nicotine-self administration in a dose-dependent manner (Scherma et al. 2016; Schindler et al. 2016). Similar data has been shown following targeting of the endogenous cannabinoid ligands (Forget et al. 2016). Stress and CB signaling have a reciprocal relationship, where exogenous CB receptor ligands increase HPA axis signaling, and antagonism of brain CRF signaling can block the increase in stress-like behaviors following CB1 receptor agonism (Puder et al. 1982; De Fonseca et al. 1996. As discussed above, the increase in stress signaling through CRF and GCs can heighten negative affective symptoms. Inhibition of the endocannabinoid fatty acid amide hydrolase exerts anxiolytic-like effects in mice (Griebel et al. 2018). Furthermore, we recently showed that cannabidiol (CBD) treatment in rats made dependent via osmotic minipump prevented both somatic signs of withdrawal and hyperalgesia during nicotine withdrawal (Smith et al. 2021). However, the mechanisms of this effect are still largely unknown as CBD is not a clear agonist of either CB1 or CB2, but may exhibit effects by binding to both receptors (Pertwee 2008). Given that CBD is used clinically to alleviate stress and pain, these data further reinforce the importance of negative affect in driving addiction-like behaviors.

## Social defeat stress and its effect on negative affect within nicotine addiction

Converging evidence shows that social defeat stress facilitates the transition to addiction through multiple mechanisms. Social defeat stress in rodents is a negative reinforcer that accelerates the acquisition of drug self-administration and triggers binge-like escalation of nicotine or psychostimulant intake (Ansell et al. 2012; Sinha 2008; Covington and Miczek 2001; Covington et al. 2005; Miczek et al. 2004; Parise et al. 2020; Zou et al. 2014; Moeller et al. 2001; Domingues et al. 2019). Social defeat stress can facilitate these symptoms, including nicotine-induced locomotor suppression (Domingues et al. 2019). Social defeat stress can also exacerbate somatic signs and anxiety-like withdrawal signs known to promote renewed consumption (Mineur et al. 2016). At the circuit level, social defeat stress increases phasic and tonic dopamine release along a VTA → accumbens → prefrontal cortex → amygdala loop and elevates CRF tone within the VTA, making rodents more prone to substance-seeking behavior (Holly and Miczek 2016; Morel et al. 2018; Ortiz et al. 2022; Adams et al. 2021; Miczek et al. 2008; Yap and Miczek 2008; Holly et al. 2016; Leonard et al. 2017; Hwa et al. 2016; Albrechet-Souza et al. 2015). Intra-VTA CRF₁ antagonism normalizes these adaptations and limit self-administration (Han et al. 2017; Burke et al. 2016). Repeated social defeat stress upregulates MOR on VTA GABA interneurons, disinhibiting dopamine neurons and deepening the shift from positive to negative reinforcement (Nikulina et al. 2008; Johnson and North 1992; Nikulina et al. 2005). Together, this dysregulation of CRF–dopamine–opioid transmissions contribute to the increased vulnerability to addition in individuals with a history of social defeat.

## Conclusions and future directions

**Fig. 3:**
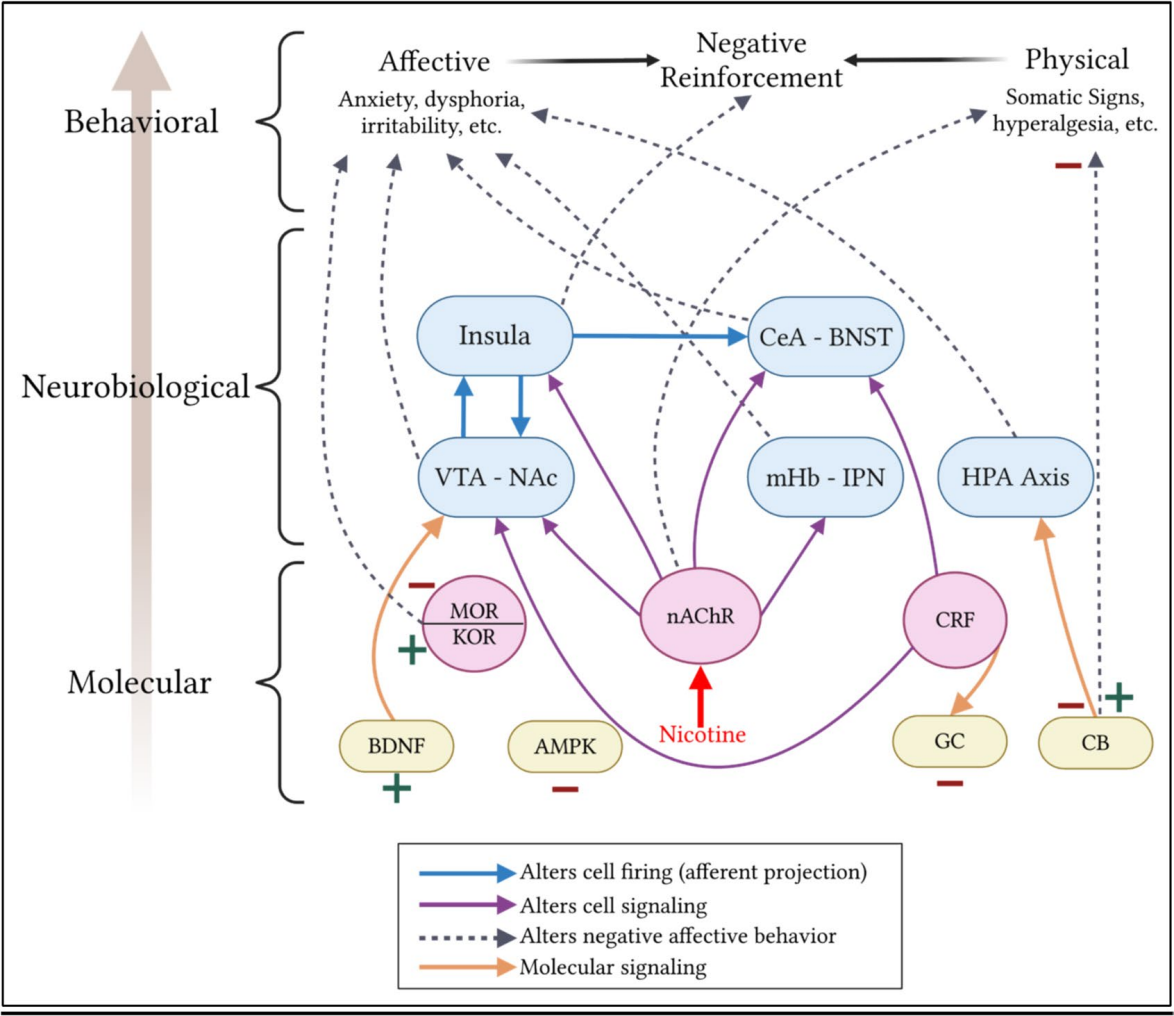
Systems-level summary of mechanisms contributing to negative affect. Signaling molecules directly impact neurobiological and behavioral mechanisms of negative affect. Nicotine binding to nAChRs influences regional and circuit signaling that subsequently alter negative affective behaviors. AMPK: Adenosine Monophosphate-activated Protein Kinase, BDNF: Brain-derived Neurotrophic Factor, BNST: Bed Nucleus of the Stria Terminalis, CB: Cannabinoid receptors, CeA: Central Nucleus of the Amygdala, CRF: Corticotropin Releasing Factor, GC: Glucocorticoids, HPA axis: Hypothalamic–Pituitary–Adrenal axis, IPN: Interpeduncular Nucleus, KOR: MOR: Kappa-Opioid Receptor, mHb: Medial Habenula, MOR: Mu-Opioid Receptor, NAc: Nucleus Accumbens, nAChR: Nicotinic Acetylcholine receptors, VTA: Ventral Tegmental Area

This review summarizes the mechanisms contributing to negative affect within nicotine addiction with an emphasis on the role of non-pharmacological stress, including social defeat stress (Fig. 3). The negative affective state emerges through the transition of nicotine taking as positive reinforcement to negative reinforcement. Negative affect and withdrawal are complex states mediated by a plethora of molecular, cellular, and circuit mechanisms to produce heightened aversive behaviors and emotional states. As nAChRs are ubiquitous within the brain, nicotine is pervasive in its reach, affecting many circuits that regulate negative affect within addiction. Importantly, stress and pain play a large role in these mechanisms. CRF is a key driver of the negative emotional states associated with nicotine withdrawal, and this is enhanced by changes in GC signaling that regulate CRF expression. Endogenous opioid signaling is also altered by nicotine and can contribute to the feelings of hyperalgesia associated with withdrawal. External stressors, particularly social defeat stress, also play a role in exacerbating drug-taking and subsequent negative affective states and withdrawal-like behaviors through altering signaling of dopamine, CRF, and opioids.

Because current therapeutic approaches for nicotine cessation are still largely ineffective (partial nAChR agonist varenicline, dopamine/norepinephrine reuptake inhibitor bupropion) (McDonough 2015), understanding not only the neurobiological drivers but also the molecular mechanisms driving negative affect can provide insight into potential therapeutic avenues to mitigate withdrawal symptoms or prevent relapse. Some current therapeutics have been shown to reduce or prevent negative affective behaviors induced by nicotine dependence; these include metabolic disease treatments metformin and GLP-1 agonists, CBD, and degradation of nicotine by NicA2. While more studies are needed to fully clarify their mechanisms of action, it expands the understanding of nicotine’s pervasive reach in altering neuronal function and signaling to potentiate its addictive effects. Of the additional signaling molecules and neuropeptides covered in this review, many are effective at reducing nicotine intake or withdrawal-like behaviors at the preclinical stage but lack efficacy at the clinical stage. CRF receptor antagonists have been explored for multiple drugs of abuse (albeit not yet successfully) as a treatment option to counter the effects of substance use on the stress system (Spierling and Zorrilla 2017), and targeting the opioid system poses its own risks in producing addiction-like behaviors. As we have shown the negative affective state to be important in the transition to negative reinforcement of nicotine use, current therapeutic strategies must take into account at least one of the following issues: reducing/preventing nicotine intake, mitigating withdrawal/negative affective symptoms, and reducing craving. Ultimately, the deeper our understanding of mechanisms driving negative affect, the greater the potential is for developing treatments to reduce or prevent nicotine addiction.

## Data Availability

All data and information presented in this manuscript are compiled from publicly available sources, thus there is no proprietary data included in this manuscript.
